# Implementing tobacco dependence treatment during clinical consultations: a qualitative study of clinicians’ experiences, perceptions and behaviours in a South African primary health care setting

**DOI:** 10.1186/1471-2296-15-85

**Published:** 2014-05-06

**Authors:** Olufemi B Omole, Olalekan A Ayo-Yusuf, Kabilabe NW Ngobale

**Affiliations:** 1 Department of Family Medicine, Faculty of Health Sciences, University of the Witwatersrand, Johannesburg, South Africa; 2 School of Health Systems & Public Health, University of Pretoria, PO Box 1266, Pretoria 0001, South Africa; 3 Director’s Office, School of Oral Health Sciences, University of Limpopo, MEDUNSA Campus, Pretoria, South Africa

**Keywords:** Healthcare provider, Behaviours, Primary care, Tobacco dependence treatment

## Abstract

**Background:**

Evidence suggests that healthcare providers (HCPs) in South Africa do not consistently offer tobacco dependence treatment (TDT) during clinical consultations. In order to understand and explain this behaviour in a South African context, we conducted a qualitative exploration of HCPs’ experiences, perceptions and behaviours regarding TDT.

**Methods:**

Individual qualitative interviews were conducted with physicians and nurses who were purposively selected. Themes were identified from interview transcripts using content analysis. Findings were triangulated and peer-reviewed, and were also verified by the participants.

**Results:**

Fifteen physicians and four nurses were interviewed, none of whom used tobacco. These participants perceived TDT as an important task, but could not consistently implement it during clinical consultations due to health systems constraints (time-constraints because of patient-overload, the unavailability of cessation medications and a lack of support for referrals), misperceptions and misconceptions (negative outcome expectations about the effectiveness and feasibility of TDT), socio-cultural barriers (counselling older persons was perceived as challenging) and personal limitations (perceived low self-efficacy, poor knowledge and skills on implementing any evidence-based TDT framework). Patients are therefore selectively screened based on clinical relevance and offered only prescriptive brief advice. Participants recommended several systems changes, including academic detailing of tobacco status, training HCPs and incorporating tobacco cessation medications in the Essential Drug List.

**Conclusion:**

The reported selective screening and limited TDT interventions offered by HCPs are related to interactions between health systems constraints, personal limitations, and misperceptions and misconceptions about the effectiveness and feasibility of TDT during clinical consultation. Implementing the recommended systems changes has the potential to improve the implementation of TDT in South African primary health care (PHC).

## Background

Tobacco use is fairly common in South Africa – according to the most recent data published based on the findings of the South African Social Attitude Survey, an estimated 33% of men and 9.5% of women were cigarette smokers in 2007 [1]. According to the same report, about 8.4% of women (predominantly black women) and 1.4% of men used smokeless tobacco, commonly in the form of snuff. Tobacco use is a significant cause of premature mortality in South Africa, accounting for 8% of deaths from all causes among people over the age of 30 years [2], and it currently costs the South African economy an estimated R1.2 billion per year to manage smoking-related diseases [3].

Despite the public health significance of tobacco use in South Africa, and the evidence that tobacco dependence treatment (TDT) provided by a health care provider (HCP) is effective in increasing quit rates [4,5], there are no dedicated tobacco cessation services available in the public health services in South Africa. Until late 2013, when a proposed clinical practice guideline was published [3], there was no standardized approach to tobacco use cessation at any clinical encounter. Most HCPs therefore relied on the limited training received at medical and nursing schools to provide TDT to their patients. Moreover, tobacco cessation medications are also not part of standard care in the Essential Drug List for primary health care (PHC). The training received by health professions students and the clinical practice guidelines promote the use of the 5As framework, but local studies suggest that South African doctors tend to miss clinical opportunities to help patients quit tobacco use [6]. For example, a recent study conducted in the current research setting found that although 26.8% of patients reported using tobacco, only 12.9% of the patients were screened, and only 11.9% of those who used tobacco were offered brief cessation advice during the clinical consultation [7]. It was noteworthy in the latter study that 88% of those who were not screened indicated they would be “very comfortable” with being screened.

Unfortunately, in view of this very limited screening and counselling behaviour, there is little South African literature to provide any in-depth understanding of HCPs’ screening and treatment behaviours. The only published local qualitative study, which was conducted in hospitals in Cape Town, concluded that doctors who provide care to pregnant women do not consider tobacco use a priority during antenatal care, nor do they intervene in their patients’ tobacco use [8]. Other studies [9-11] that provide insights into HCPs’ behaviours during the clinical consultation were mostly conducted in contexts that differ from those in South African PHC. This makes transference and the application of findings into the South African PHC setting a challenge.

Considering the lack of studies on HCPs’ behaviours in South African literature and the findings of the previous quantitative study in the current research setting, which suggests that only about one in ten patients is screened, and that only a similar proportion of those screened are actually advised against tobacco use, the aim of this qualitative study was to gain an in-depth understanding of HCPs’ behaviours by exploring their experiences, perceptions and behaviours regarding the implementation of TDT during clinical consultations. It was hoped that in providing an understanding of HCPs’ behaviours, this study could assist in optimizing the implementation of TDT in clinical consultations in South African PHC or in similar contexts.

## Methods

This research was a qualitative study of HCPs conducted in two Community Health Centres, south of Johannesburg, South Africa. A qualitative design was employed, because an in-depth understanding of HCPs’ lived experiences, perceptions and behaviours can best be achieved using qualitative methods [12]. At the time of this study in 2010, each Community Health Centre provided comprehensive PHC services to over 6000 adult patients per month via five PHC nurses and any three of the eighteen available physicians in the district. Patients are triaged in such a way that the less complex clinical cases are attended to by PHC nurses, while physicians attend to more complex cases. Both Community Health Centres form part of an academic complex for postgraduate Family Medicine and the training of clinical PHC nurses.

All physicians and PHC nurses in the two facilities were regarded as key informants and were purposively invited to participate in the study through the office of the facility managers. Ultimately, fifteen physicians and four nurses were interviewed, based on their response to the invitation and the attainment of theoretical saturation during interviews. The researchers decided to stop further recruitment when they were satisfied that theoretical saturation had been attained with the nineteenth participant’s interview.

At the appointed times for the interviews, written informed consent was obtained from each participant and each participant completed a demographic and tobacco use questionnaire. A trained research assistant who holds a diploma in commercial education conducted the one-on-one interviews, which were audio-taped and supplemented with field notes. In addition to prior interviewing experience, this research assistant received training in qualitative methods and interviewing techniques. The interviews all began with an exploratory question: “***What is your experience of implementing tobacco cessation counselling during primary care consultations, if any?****”* Interviews were facilitated using open-ended questions, reflections and summaries, guided by an interview schedule. Each interview was transcribed verbatim by the interviewer afterwards, and the transcript was supplemented with information from the field notes.

Themes were independently identified from the transcripts by the first and third authors using content analysis. The findings were validated against an analysis by an independent peer who was not part of the research team. The themes identified by the three analysts were compared and triangulated against each other to achieve consensus. Where there was disagreement, a re-analysis was done until consensus was reached. Similar themes were aggregated into categories in a group exercise by the three analysts. The combined themes were then subjected to peer-review by the second author, who acted as a “devil’s advocate” by critically appraising the themes and the meanings ascribed to them to ensure they are true findings in the context of the study. The study findings were taken back to four of the nineteen participants (three physicians and one nurse) for validation, and they confirmed that the findings were a true reflection of their perceptions.

Ethics clearance and permission for this study were obtained from the Human Research and Ethics Committee of the University of Witwatersrand [*Number: M080513*] and the Sedibeng Health District Management.

## Results

Of the nineteen participants interviewed, four were nurses and fifteen were physicians (Table 1). All participants were Black Africans, except for one expatriate Cuban physician. Six major themes were identified from the interviews. These are listed below and are then discussed, reflecting verbatim comments in italics.

1. HCPS’ knowledge and perceptions of their role(s) in implementing TDT

2. Screening and counselling behaviours

3. Feasibility and barriers to implementing TDT

4. Awareness of the 5As tobacco cessation framework

5. Perceived public health implications of tobacco use

6. Perceived need for a system change

**Table 1:** Participants’ demographic and tobacco use characteristics

**Characteristics**	**Frequency % (n)**	**Mean**
**Age (yrs)**		39.6 (Range: 26–56)
**Sex**		
Male	9	
Female	10	
**Clinician’s category**		
Doctor	15	
Nurse	4	
**Marital status**		
Single	4	
Married	12	
Divorced	1	
Widowed	1	
Separated	1	
**Years of experience in PHC**		4.5yrs (Range: 3 months–18 yrs)
**Tobacco use status**		
Current user	-	
Previous user	1	
Never used	18	

### Description of themes

1. **HCPS’ knowledge and perceptions of role(s) in implementing TDT**

Participants perceive TDT as a useful intervention, in that it can have a favourable impact on the quality of health care: “*Tobacco counselling is useful in a sense that it helps to improve the quality of our service. .....so we know that the cessation of tobacco has good impact on life in the future ***(P7; Physician, <5 years’ experience)**. Participants also reported that their knowledge of the dangers of smoking promotes their engagement in TDT. Participants identified their role in assisting patients to stop tobacco use and preventing other patients from starting: *“I will always endeavour to help people to stop smoking, counselling the people not to start smoking because when they start, it is very difficult to quit ***(P15; Physician, >5 years’ experience)**. Another participant stated that *“some of them [tobacco users] have the will to stop but don’t know how and.....don’t have enough support. So.....counselling has to be given” ***(P16; Physician, <5 years’ experience)**. While initiating TDT is perceived to be the clinician’s responsibility, patient readiness to receive advice was also perceived to play a crucial role: *“…so we give the information and leave it, because they are adults, they know what to do.....and we cannot force it on them ***(P6; Nurse, <5 years’ experience)**.

2. **Screening and counselling behaviours**

HCPs do provide TDT, although they do so in a selective manner. The screening and counselling behaviours of HCPs are influenced by several systems and HCPs’ attitudes. These behaviours are described further under the subthemes below.

• **Selective screening based on patient characteristics:** Participants selectively screen patients due to time constraints, and provide counselling to patients based on perceived clinical relevance: *“We are having high turnover [of] patients, unfortunately our time is limited, but when we come across a need [relevance], we are able to provide counselling to patients on quitting smoking and adverse [effects] of cigarette use [on] our patients, specifically asthmatic, hypertensive and diabetic patients” ***(P1; Physician, <5 years’ experience)**. Another participant reported that *“…those [patients] who ask for help, are receptive or show tobacco-related problems or chronic conditions always get my full attention” ***(P5; Physician, >5 years’ experience)**. The presence of physical signs of smoking (tobacco odour, nicotine stains on moustache and nails, etc.), chronic diseases, parents of small children, and women in the reproductive age group are considered cues to action and tend to prompt tobacco cessation interventions. Men and older patients tend to be resistant, and race also influence counselling*: “…we are having problems with Whites; they will tell you that even with my first pregnancy I was smoking and my baby is healthy .... So, the Blacks are the ones who listen and adhere to what I taught them” ***(P4; Nurse, <5 years’ experience)**. Trans-generational resistance also influences the behaviour of HCPs and is evident in the following statement*: “So it depends on what kind of patient. You may find that [if] it is a younger patient, usually it is difficult to say to them they must stop smoking because they think it is the ‘in thing’ to do. …Then the older patients, you may find that they will tell you that they have been smoking for a long time…so it influences me ...., because they don’t want to listen when it comes to smoking” ***(P12; Physician, <5 years’ experience)**.

• **Limited offer of TDT:** Although participants offer some form of assistance, most do not go beyond providing prescriptive brief advice (asking patients to stop smoking) to tobacco users*: “..****.****.but we don’t really go into depth…because it is very difficult if you see [attend to] 72 patients per day…” ***(P8; Nurse, <5 years’ experience)**. However, one participant reported counselling and assisting patients, influenced by the training the participant had received: *“....until recently when I started doing my [Family medicine] residency programme, I started reading widely about this thing [tobacco cessation]....I have started to ask patients not just to stop [smoking] but sometimes I even go further to counsel them. When they are prepared to stop, sometimes we have to use drugs…like nicorette gum....” ***(P10; Physician, >5 years’ experience)**.

• **Lack of continuity of care:** This makes follow-up of tobacco users difficult: *“The experience is that you can see the patient today and tomorrow [the] patient is seen by my colleague. It is difficult to do follow up… ***(P2; Physician, <5 years’ experience)**. However, monthly appointments and documentation of tobacco use status promote follow-up among pregnant women: *“In antenatal, we do follow-ups monthly and if we see on the cards that [they] are smokers, because we have to write it on the cards, then we do follow-up, ….” ***(P8; Nurse, <5 years’ experience)**.

• **Influence of HCPs’ characteristics on screening and counselling behaviours*:*** Although both the physicians and the nurses reported implementing TDT, the nurses are perceived to do more counselling*: “ I am aware that PHC nurses do a lot of counselling with regards to adverse [effects] of smoking and how to quit and what measures to take and how to check and make follow ups with them” ***(P1; Physician, <5 years’ experience)**. Frequent encounters with patients presenting with smoking-related diseases prompt some HCPs to implement TDT*: “…seeing lots of patients with diseases caused by smoking… would encourage me to practice tobacco counselling” ***(P12; Physician, <5 years’ experience)***.* However, implementation of TDT is less likely when there is a disparity between the HCPs and the patient’s age, as it may have a negative cultural impact on the way counselling is perceived: *“......it is a big issue to me, like now you are asking your own father to stop smoking...... and if you know ‘ubuntu’ [African worldview of “I am what I am because of who we all are”]....he might look and [think] does this guy have respect?” ***(P10; Physician, >5 years’ experience)**. Participants’ personal dislike for smoking promotes their engagement in TDT, but their perceptions of low self-efficacy limit the extent of their engagement.

3. **Feasibility and barriers to implementing TDT**

Most participants perceive that TDT is not feasible within the clinical consultation because of time constraints, competing tasks in the consultation, their poor counselling skills and the unavailability of cessation drugs: *“…it is not really practical because [of what] I told you before – the time. We have to deal with so many things…” ***(P13; Physician, <5 years’ experience)**. Another participant commented: *“…At the moment,.....we don’t have any special counselling abilities [with which] we can....encourage them [patients] to stop smoking…” ***(P1; Physician, <5 years’ experience)**.

Other participants, however, perceive TDT to be feasible in a clinical consultation, especially when patients are briefly advised and referred to specialized centres for further support and treatment: *“[It] is feasible in the sense that it is does not take long, it takes two, three or ten seconds…You can cover it within a minute, at least touch on it and then refer the patient” ***(P16; Physician, <5 years’ experience)**.

4. **Awareness of the 5As tobacco cessation framework**

Most participants were not fully aware of the 5As framework, but use self-knowledge to deal with tobacco use in the consultation: *“I won’t say I am aware of all the 5As. I know about ‘ask’ and ‘advise’, and then as for follow [up],........I don’t” ***(P12, Physician, <5 years’ experience)**. This lack of awareness is due to a lack of training in tobacco cessation: *“No I haven’t undergone any formal or informal training, but yes I just use my own knowledge of what the dangers of tobacco use (are)....” ***(P12, Physician, <5 years’ experience)**. Undergoing training is therefore perceived to be important: “*I would say it [training] is necessary because we never smoked before. It is difficult for us to tell somebody to stop, if you haven’t been in that position before” ***(P8; Nurse, <5 years’ experience)**. Family medicine trainees appear to be more aware of the details of TDT and the skills to perform them: *“…we [are] taught about counselling and we know how to do it ........at least we had some lectures on counselling” ***(P13; Physician, <5 years’ experience).**

5. **Perceived public health implications of tobacco use**

Participants reported that although the burden of smoking is high and the resulting addiction is strong, they did not think anti-tobacco programmes were a priority on the government’s agenda: *“....if you check our [health] calendar, there is not a lot about tobacco or smoking, it is only HIV [the Human Immunodeficiency Virus], STI [sexually transmitted infections] and TB [tuberculosis], and the thing that causes TB in most cases is smoking” ***(P4; Nurse, <5 years’ experience)**. The participants themselves do not perceive tobacco use in itself as a health problem, unless it is related to a presenting clinical problem, and they believe that most reported health implications relate only to smoking and not to other forms of tobacco use. Men are perceived to smoke, while women use snuff: *“Most of the patients [smokers] that I get are men but the females are using snuff” ***(P17; Physician, >5 years’ experience)**. Although “Youth Friendly Services” are available in clinics, these are perceived not to address tobacco use among young people. Tobacco use is also perceived to have financial implications as it may divert money from being used on basic necessities: *“....money that could have been used to buy food and other things are being directed to buy tobacco and cigarettes…” ***(P10; Physician, >5 years’ experience)**. HCPs are also perceived to be disease-focused and as not exploring the socio-economic impact of tobacco use.

6. **Perceived need for system change**

Participants’ suggestions regarding changes to improve the implementation of TDT include:

• TDT should be done routinely, seeing tobacco use as a form of vital sign: *“It is very important especially to counsel patients on tobacco use routinely. I went to a course where it was said that we need to incorporate it, like in every consultation.......it must become like a routine vital sign to check smoking habit” ***(P11; Physician, <5 years’ experience)**.

• Participants acknowledge that counselling alone is not effective and that additional interventions are needed. It was recommended that tobacco cessation drugs be incorporated into the Essential Drug List. Treatment and referral support networks, which include a national tobacco quit line, should be established.

• Tobacco should be *“banned completely [from] the market, taking into consideration that it doesn’t give us benefits. It causes lung diseases, hypertension, diabetes, chest pain…” ***(P13; Physician, <5 years’ experience).**

• Government should be more involved by developing policies and raising awareness through campaigns, ”…*just like HIV campaign that is going on…” ***(P14; Physician, >5 years’ experience)**. These anti-tobacco campaigns should be extended to churches, schools and workplaces, using multiple campaign media.

• Training should be provided for HCPs: *“…it is really necessary to attend training. It will help me to manage the patients…” ***(P17; Physician, >5 years’ experience)**.

**Figure 1:**
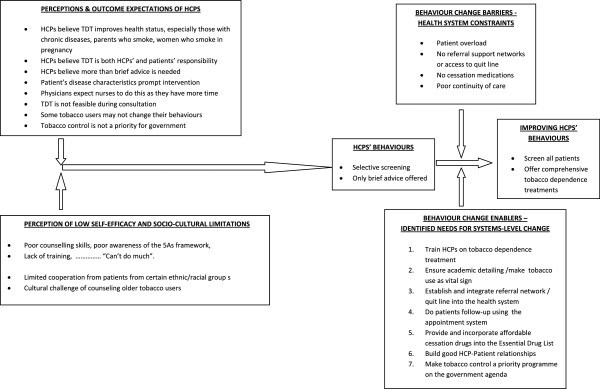
Emerging explanatory model consistent with social cognitive theory.

Put together, all the findings of this study are consistent with the framework of social cognitive theory (Figure 1), which posits that human behaviours derive from interactions between personal, cognitive and environmental influences and experiences [13,14].

## Discussion

This study found that participants perceive the implementation of TDT to be an important and beneficial task, and that HCPs’ behaviours result from their perceptions of their roles in implementing TDT, their knowledge and counselling skills (or lack of such skills), misperceptions and misconceptions about the effectiveness and feasibility of TDT during the consultation, and several limitations imposed by the health system (time constraints arising from an excessive patient load, a lack of continuity of care and the unavailability of cessation treatment modalities). While similar findings have previously been reported in studies conducted elsewhere [8,15-17], this study provides the first in-depth understanding of HCPs’ behaviours in South African PHC and suggests a need for multipronged interventions to improve the implementation of TDT.

### HCPs’ perceived role(s) and selective screening behaviours

Participants recognized and accepted their role(s) in assisting tobacco users to cease tobacco use, but time constraints imposed by high patient loads were probably responsible for their opting for selective screening and for counselling based on clinical relevance. HCPs may attempt to maximize the impact of TDT by focusing only on patients with diseases perceived to be tobacco-related [18,19]. Nonetheless, the HCPs did not always perceive tobacco use as a health problem, except when it is associated with a disease state. Patients who do not present with physical ailments are therefore not likely to be screened. Such selective screening behaviours result in missed opportunities for TDT, which can be addressed by adopting tobacco use status as a vital sign. This has been shown to improve screening, counselling and referral rates [20-22].

In South Africa, physicians and PHC-trained nurses render clinical services. Although both cadres of HCPs accepted responsibility for implementing TDT in this study, in the South African PHC context, physicians take on more complex patients compared to PHC nurses, and, in the context of heavy patient loads, this may result in doctors’ relegating their counselling role to nurses. Nurses are therefore perceived to “do a lot of counselling”. However, physicians have also been known to delegate such tasks in the belief that smoking cessation assistance may be best provided by other categories of HCPs [23]. The latter may be the more likely explanation for physicians’ failure to provide TDT in this study, given that most physicians’ perceived themselves as not knowledgeable and as having low self-efficacy with regard to TDT.

### Misperceptions, misconceptions and poor knowledge of the cessation process

HCPs’ behaviours are influenced by several misperceptions and misconceptions. Misperceptions that HCPs should only “…*give the information and leave it, because they are adults, they know what to do.....and we cannot force it on them*”, and that it is the patient’s prerogative to stop smoking may signify HCPs’ lack of knowledge and understanding of the entire TDT process. Not understanding nicotine addiction and the process of cessation could result in prejudices, negative attitudes and missed opportunities, with the result that patients who need additional interventions beyond offering advice are not assisted. It is known that most tobacco users want to quit [24], but chemical dependence and withdrawal symptoms make it an uphill task [25], indicating that most tobacco users will require support in some form for a successful outcome. Considering that patients do not always make a change to less risky behaviours at one go (even in the face of a serious diagnosis) [26], HCPs need to recognize their duty to motivate tobacco users to quit and to continue to provide support to patients during the process of changing undesirable health behaviours [17]. This is especially important in South Africa, where reports suggest that doctors do not take responsibility for, nor intervene in, their patients’ tobacco use habits [8].

Despite evidence that behavioural therapy alone, including brief advice provided by an HCP are effective in promoting tobacco use cessation [6,27], participants in this study reported that counselling is not effective on its own, except when combined with cessation drugs. This misconception may reflect one or more of three issues: HCPs’ lack of awareness and understanding of the effectiveness of counselling as a stand-alone intervention, their negative outcome expectation of tobacco cessation counselling, or their perceptions of low self-efficacy in providing TDT [8,28]. These misconceptions create an inertia that hinders the use of brief advice and varying levels of counselling intensity as effective interventions, and signify an urgent need for training. Although tobacco cessation drugs facilitate cessation [29], they should not preclude counselling. Moreover, considering that most South African smokers are light smokers [29], they can be assisted to quit with appropriate counselling and behavioural support.

Participants’ poor awareness of the 5As framework or any other evidence-based approach is a reflection of their lack of knowledge and skills, and may explain their perceived low self-efficacy in TDT. Saloojee and Steyn [6], explain that the reluctance of doctors to act and assist tobacco users points to a lack of skills and competence [6]. A similar reflection of lack of knowledge is the fact that the participants in the current study focused mostly on the adverse health effects of smoking and did not report the health implications of snuff use, second-hand smoke and other forms of tobacco use, though these are also associated with significant morbidity and mortality [30,31]. Therefore HCPs need to be trained on the health implications of all forms of tobacco use and on the need to screen for them, including screening for other addictions, given that concurrent addictions decrease the likelihood of successful quit attempts [29,32]. Training has been shown to increase providers’ knowledge and confidence, as well as the likelihood and the extent of implementing tobacco cessation interventions [17,20,33-36]. In order to address this knowledge gap through training, Saloojee and Steyn [6], suggest that for HCPs to recognize and take advantage of the opportunities provided by the clinical encounter, changes in medical school training are required [6]. Their proposal will equip health professions students to prepare for clinical work, but currently practising HCPs who are products of deficient curricula will also require structured and ongoing in-service training to improve their competence in TDT.

Counselling is effective as a tool for effecting behavioural change [26]. However, African cultural views such as that expressed by one participant (**P10; Physician, >5 years’ experience**) on how people of different ages should relate could pose a challenge to counselling against tobacco use. The view that young HCPs may be seen as disrespectful when they try to motivate older patients to quit tobacco use implies that HCPs should be culture-sensitive in their approach to tobacco cessation counselling [26,37]. Similarly, although there is no evidence to suggest this is a systemic problem, the difficulties experienced by some HCPs and patients regarding different races in the giving and receiving of health advice may indicate remnant historical prejudices in post-apartheid South Africa, and calls for a response that does not constitute further barriers to implementing TDT during a clinical encounter.

### The need for system changes

Several of the participants’ perceptions highlight a need for changes in the way TDT is currently implemented. Suggested changes are discussed below.

Firstly, in order to minimize missed opportunities for tobacco prevention, tobacco use status needs to be adopted as a vital sign. This would ensure that all patients are screened. Indeed, maximizing the impact of TDT requires HCPs to screen all patients and provide treatment support to all tobacco users [5]. This may not be always feasible in a setting where there are extreme time constraints, but providing treatment opportunities, particularly referral support networks to which tobacco users can be referred may result in more quit attempts and higher quit rates [5]. Such support networks should also be used as points of contact for follow-up in order to address the reported lack of continuity of care. Surprisingly, most participants were not aware of the existing national quit line, which reveals the need to integrate quit lines formally into health services in South Africa.

Secondly, most HCPs lack knowledge, are poorly skilled, and have misconceptions and misperceptions about TDT. This indicates an urgent need for HCPs to be trained on TDT. Such training should aim to provide information that dispels myths, misconceptions and misperceptions, and should also be leveraged on the fact that the majority of HCPs do not use tobacco themselves to promote the implementation of TDT by them, as this cohort is more likely to engage in TDT [38].

Thirdly, there is abundant evidence of the effectiveness of tobacco cessation medications [39]. Many tobacco users will quit with counselling and behavioural support, and the addition of cessation medications to counselling when indicated is likely to increase the odds of successful cessation [4]. There is therefore a need to incorporate them into the Essential Drug List at the PHC level in South Africa.

Fourthly, the perception that tobacco control is not a priority on the government’s agenda may explain participants’ experiences of a lack of organizational support for tobacco cessation treatment in their own setting. Considering that tobacco use is an important risk factor for morbidity and premature mortality in South Africa [2], TDT in the clinical setting needs to be prioritized on an equal footing with other health programmes such HIV/AIDS and TB programmes, in line with the government’s commitment to the recent United Nations declaration on the eradication of non-communicable diseases [40].

Fifthly, participants’ recommendation that anti-tobacco campaigns be expanded to community organizations using multimedia may be informed by a perceived need for an expanded and innovative anti-tobacco campaign platform. Such innovative approaches to anti-tobacco campaigns that take advantage of developments in information technology and advertisement media can extend the reach of anti-tobacco campaigns. Admittedly, while innovative campaigns may be well received especially by the young, the evidence that they increase cessation rates remains inconclusive [41].

Lastly, the view that tobacco diverts money from essential materials of life and the suggestion that HCPs need to explore the socioeconomic effects of tobacco use have been confirmed in reports that found that tobacco use is a “financial drain” and is associated with poverty [42]. Given that poor people are most sensitive to the price effects of tobacco, future studies need to explore the cost implications and socioeconomic patterning of tobacco, including the economic gains of tobacco control in South Africa.

### Explaining HCPs’ screening and counselling behaviours using the social-cognitive theory

An explanatory model (Figure 1) based on social cognitive theory was developed to explain the interconnectivity of themes identified in this study. The social cognitive theory explains how behaviours are acquired and maintained, and proposes that behaviour change result from continuous interactions between cognitive, personal and environmental factors [12]. These factors have varying strengths in interactions and do not necessarily occur concurrently [13].

In this study, it is proposed that the participating HCPs’ behaviours resulted from continuous interactions between environmental and socio-cultural factors (healthcare system demands and constraints, and patient characteristics), personal cognitive factors (poor outcome expectations of HCPs' behaviours, misperceptions and misconceptions regarding the effectiveness and feasibility of TDT, HCPs’ lack of adequate knowledge and skills) and perceptions of low self-efficacy in offering TDT). These complex interactions inform HCPs’ decisions not to screen all patients, but to selectively screen patients with tobacco-related diseases.

HCPs’ tobacco use screening and treatment behaviours are indeed influenced by the demands and limitations imposed by local healthcare and social systems. The demand to implement TDT is exemplified in perceptions that it is important to implement TDT to improve the health status of patients and that it is an HCPs’ responsibility to implement TDT. On the other hand, perceptions that tobacco control is not a priority on the government’s agenda, perception that TDT is not feasible within consultations happening under pressure in overcrowded clinics, a lack of treatment and referral support, the unavailability of cessation medications and negative cultural views, all limit full implementation of TDT.

HCPs’ perception and misconceptions about their own self-efficacy and the effectiveness and feasibility of TDT also interact with other factors to determine HCPs’ behaviours. For example, most HCPs have not been trained in, and therefore lack knowledge and awareness of, any evidence-based tobacco treatment framework, which in turn results in poor counselling skills and low self-efficacy. Given an environment characterised by high patient loads and no referral support, HCPs hold a perception that “much can’t be done”. In addition, there are perceptions that counselling is not effective on its own, that it needs to be combined with medications, and there is an outcome expectation that patients may not change their behaviours even when counselled – all this results in most HCPs not screening and advising tobacco users to quit.

Participants recommended several system changes aimed at changing HCPs’ behaviours. In the proposed model, if the government were to prioritize tobacco control in a clinical setting, then training of HCPs would become a priority. Training can boost HCPs’ self-efficacy, which in turn alters HCPs’ outcome expectations so that HCPs may perceive it to be possible for tobacco users to change their behaviours when they are helped. Establishing referral support centres and quit lines to which tobacco users can be referred (or making HCPs aware of existing structures of this nature) can also alter HCPs’ current outcome expectations of the feasibility of TDT within the clinical consultation to a favourable one. These changes in outcome expectations can in turn promote screening of all patients and the provision of TDT. Adding cessation drugs to the Essential Drug List may increase HCPs’ confidence in their ability to intervene, knowing that if tobacco users fail to quit with counselling, cessation medications can be added, a practice which has been shown to be more effective than counselling alone [43]. Implementing these recommendations could alter this triadic model and favourably change HCPs’ behaviour to increase screening and offering comprehensive TDT.

### Limitations

This study used a qualitative design and the findings may therefore not be generalized. The study was also based on participants’ self-reports, making the findings susceptible to information bias. The position of two of the researchers (senior clinicians) in the research setting could have unduly influenced the responses of the participants. However, self-awareness of the researchers, the use of facility managers as recruiters, the use of a trained research assistant for the interviews and the methodological rigour employed in assuring credibility and trustworthiness, all assisted in limiting potential undue influences and biases.

## Conclusion

Participants perceived TDT to be an important and beneficial task during a consultation, but indicated that they applied selective screening and counselling behaviours as a consequence of interactions between healthcare systems constraints, their own limited knowledge and skills, and misperceptions and misconceptions about the effectiveness and feasibility of TDT.

Implementing systems changes such as institutionalizing tobacco use as a vital sign, providing training to HCPs, including cessation medications in the Essential Drug List, integrating quit lines into PHC, sustained anti-tobacco advocacy campaigns, and leveraging TDT on HCPs’ non-tobacco use status, all have the potential to improve the implementation of TDT during the consultation in South African PHC.

## Abbreviations

HCP(s): Health care practitioner(s); PHC: Primary health care; TB: Tuberculosis; TDT: Tobacco dependence treatment.

## Competing interests

The authors declare that there are no financial or personal relationships that may have inappropriately influenced the study.

## Authors’ contributions

OB was involved in the conception, design, data analysis and interpretation of the results, drafting and revision of the manuscript, and has given approval for this version of the paper to be published. OA was involved in the conception, design, data analysis and interpretation of results, revision of the manuscript and has given final approval for the final version to be published. KNW was involved in the conception, design, data analysis and interpretation of results and has given approval for the final version to be published. All authors read and approved the final manuscript.

## Authors’ information

OB is Head of Clinical Unit (Family Medicine) at Sedibeng District Health Services, Gauteng Department of Health, South Africa, in joint appointment with the Department of Family Medicine, University of the Witwatersrand, South Africa.

OA is an Extraordinary Professor in the School of Health Systems & Public Health, University of Pretoria, and a Professor and Director of the School of Oral Health Sciences at the University of Limpopo, MEDUNSA campus, South Africa, in joint appointment with the Gauteng Department of Health, South Africa.

KNW is a Specialist Grade 2 (Family Medicine) at the Sedibeng District Health Services, Gauteng Department of Health, South Africa, in joint appointment with the Department of Family Medicine, University of the Witwatersrand, South Africa.

## Pre-publication history

The pre-publication history for this paper can be accessed here:

http://www.biomedcentral.com/1471-2296/15/85/prepub
